# Failed Examinees' Legal Challenge over the Clinical Skill Test in the Korean Medical Licensing Examination

**DOI:** 10.3352/jeehp.2010.7.5

**Published:** 2010-12-05

**Authors:** Sun Huh

**Affiliations:** Department of Parasitology, College of Medicine and Institute of Medical Education, Hallym University, Chuncheon, Korea.

The first trial of the clinical skill test for the 2009 Korean Medical Licensing Examination was completed without remarkable problems or mistakes. In this volume, a technical report by Dr. Kun Sang Kim, President of the National Health Personnel Licensing Examination Board (NHPLE) of Korea describes the introduction and administration of the clinical skill test [1]. However, afterward, 66 failed examinees filed a legal dispute. In February 2010, they sued the NHPLE to cancel their failure of the clinical skill test based on three justifications. First, there was variation in test item difficulty because the items were different for each group. Second, raters of the clinical performance examinations were standardized patients, not medical doctors like the objective structured clinical examination. Third, unlike the written test, there was no announcement on the passing criteria before the test [2]. Those three issues had already been taken into consideration and the NHPLE felt it had addressed them adequately by adopting the testing system of foreign countries where there is longer history of clinical skill testing than that of Korea. The judge is going to decide the case in early December 2010. The results of this case may influence further execution of Korea's clinical skill test.

The NHPLB has encountered similar cases every year it has administered the licensing examination because each time a number of examinees have not accepted the results of the test. It is the right of examinees to voice their concerns. However, medical health personnel are professionals. Therefore, the dispute would be better resolved through the professional societies if at all possible. This would allow the medical health profession to retain a public face of dignity and authority. As for medical education and its evaluation, medical faculty members are professional specialists. The NHPLB's quality has been maintained by volunteer professors' devotion and sacrifice. Although it is not perfect, the present system is the best possible outcome after long deliberation and research. If there are any weaknesses in the examination, they should be overcome by ongoing study and communication. The culture of lawsuits has become more pervasive in Korea in every field nowadays. This may reflect the increase in more reasonable dispute-solving behavior and the ease of bringing lawsuits due to low legal fees. Nevertheless, it is necessary to minimize the possibility of trivial lawsuits while still allowing for test takers to voice legitimate concerns to prevent the waste of resources. From now on, a method of preventing such trivial lawsuits against the NHPLB should be developed and examination candidates should be informed of the updated procedures through the medical health field institutes. In short, a new system to meet objections to examinees' results should be developed.
